# A Comparative Study of Standard and Direct Susceptibility Testing From Positive Blood Cultures at a Tertiary Care Hospital in Bangladesh

**DOI:** 10.7759/cureus.96110

**Published:** 2025-11-04

**Authors:** Ishad Mazhar, Sofia Andalib, Rumana Alim, Shaila Munwar, Sharmin Khan, Raisa Badhan

**Affiliations:** 1 Microbiology, Medical College for Women and Hospital, Dhaka, BGD; 2 Microbiology, National Institute of Burn and Plastic Surgery, Dhaka, BGD

**Keywords:** antimicrobial susceptibility testing, blood culture, direct susceptibility test, disk diffusion, septicemia

## Abstract

Background: Sepsis is one of the leading causes of death globally. Standard antimicrobial susceptibility testing (AST) takes around 48 hours to get results from a positive blood culture bottle. Advanced diagnostic tools for AST are not available in most of the laboratories in developing countries. Therefore, performing a direct susceptibility testing (DST) might help to reduce turnaround time by 24 hours, reducing high mortality in patients with septicemia.

Objective: The study aimed to evaluate the accuracy of DST from positive blood culture broth by comparing categorical agreement and errors against the standard AST of the isolates.

Methods: Blood culture bottles flagged as positive by the automated system between January 2024 and December 2024 in a tertiary hospital in Bangladesh were included in this cross-sectional study. Identification of organisms and AST were performed using both DST and standard AST methods, according to the Clinical and Laboratory Standards Institute’s guidelines. The results of the two methods were compared for agreement or errors.

Results: A total of 100 bacteria were detected by DST and standardized AST method from the positive blood culture bottles, where* Salmonella typhi *(38%) was the most prevalent Gram-negative bacterium, and coagulase-negative *Staphylococci *were the most common Gram-positive bacteria (26%). When compared to the standard AST, categorical agreements of DST were > 93% for *Staphylococcus *species, *Salmonella typhi*, *Escherichia coli*, *Klebsiella*, *Enterobacteri*, and *Pseudomonas *species. The overall categorical disagreements for these organisms were within acceptable limits.

Conclusion: DST from positive blood culture broth can reduce turnaround time and enable earlier initiation of antibiotic therapy in septicemic patients.

## Introduction

Bacterial bloodstream infections can lead to life-threatening sepsis, and it is one of the most important causes of morbidity and mortality worldwide [1]. Each hour of delay in administering antibiotics increases the mortality rate in patients with septic shock. Therefore, the early and accurate determination of the antimicrobial susceptibility of bacteria is essential for ensuring the treatment of patients with sepsis [1,2]. Early availability of sensitivity reports not only helps the clinician to reduce the use of antimicrobials but also lowers the chances of the emergence of resistant organisms [3]. To date, blood culture is the gold standard method that can help in the identification of microorganisms and antibiotic susceptibility testing (AST) for the diagnosis and treatment. Furthermore, integrating rapid tests to detect AST can significantly improve the availability of results with targeted therapeutic approaches [4].

The increasing rates of antimicrobial resistance (AMR) demand the development of rapid diagnostic tools for AST. Automated/semi-automated devices and molecular methods offer fast and reliable microbial identification and AST with high specificity and sensitivity [5]. While advanced diagnostic tools for AST can be effective, where antibiotic resistance is high, they present substantial challenges. The efficacy of advanced diagnostic tools for AST is acknowledged; however, a significant challenge arises from the high cost and limited accessibility of resources in low-to middle-income countries. Therefore, identification of bacteria is done by traditional subculture, biochemical testing from positively flagged bottles of automated systems like BacT/ALERT or BACTEC, and routine AST is done by the Kirby-Bauer disk diffusion method [6].

The standard routine method for positive blood cultures involves the subculture of blood culture broth onto agar media plates. Plates are then incubated overnight aerobically at 37°C to obtain isolated colonies. Gram staining and biochemical tests are done on isolated colonies. A standardized inoculum is made from these colonies, which is used for routine AST [1,3]. It takes more than 24-48 hours to get an antibiotic susceptibility report from the time of the positively flagged blood culture bottle by the routine method. So, early administration of the appropriate antibiotics is difficult [2]. To reduce the total turnaround time of the current antibiotic susceptibility detection process, various methods have been developed. The disk diffusion method, directly from positive blood culture rather than isolated colonies, saves an additional 24 hours and emerges as a highly practical and cost-effective alternative [5-7]. Thus, obtaining results 24 hours earlier than the routine AST is possible. Direct susceptibility testing (DST) is a well-established diagnostic work-up of bloodstream infections from positive blood culture broths. Shortening the time to get results for susceptibility testing of blood culture isolates can significantly reduce patient morbidity and mortality by initiating earlier targeted antibiotic therapy. This method is simple to implement, especially when compared to more expensive diagnostic approaches. Moreover, rapid reporting of susceptibility results derived from disk diffusion of positive blood culture broths has been demonstrated to decrease length of hospital stay and lower healthcare costs [8-10]. Multiple comparative studies have been conducted in different countries in Asia, like India, Pakistan, and Hong Kong, to detect the reliability of the DST. These studies evaluated the effectiveness and limitations of DST by comparing results against standard AST [11-14]. However, limited data regarding the accuracy of DST are available in Bangladesh. This study aimed to assess the accuracy of DST compared to the standard AST for early bacterial identification and selection of appropriate antibiotics in clinical settings.

## Materials and methods

This comparative analytical laboratory study included a total of 100 positively flagged blood culture samples between January 2024 and December 2024. Laboratory procedure was conducted at the Department of Microbiology, Medical College for Women and Hospital.

Sample size calculation was done by considering the prevalence to be 50% with the formula of the prevalence of sample size calculation. Study samples were blood culture bottles flagged as positive by the BACTEC system.

Inclusion criteria

Blood cultures with a positive signal flagging off from the automated blood culture system show only one type of organism by direct smear examination.

Exclusion criteria

Polymicrobial growth or more than one type of organism by direct smear examination was excluded from the study.

Laboratory procedure

DST (Test Method)

After the blood culture bottle was flagged positive by the BD BACTEC FX40 system, four drops of blood were dispensed onto Mueller-Hinton Agar (MHA) using a sterile syringe [15]. The blood was spread on the MHA plate with a cotton swab, and antimicrobial disks were applied for both Gram-positive and Gram-negative organisms. Concurrently, subcultures were made on blood agar, MacConkey’s agar, and xylose lysine deoxycholate (XLD) agar, along with biochemical tests (Kligler Iron Agar, Simmon's citrate agar, motility, indole, urease, oxidase, catalase, and coagulase test) being performed. Kligler Iron Agar, Simmon's citrate agar, and motility, indole, and urease tests were performed from broth, and oxidase, catalase, and coagulase tests were done from colonies or sensitivity plate. Gram staining was performed on the blood broth. All culture plates and media were incubated aerobically at 37°C overnight. The following day, isolates were identified via biochemical test results, and susceptibility reporting was completed approximately 24 hours after the blood culture bottles were initially flagged as positive.

Standard AST (Reference Method)

Standard AST results were used as a reference and were performed using the Kirby-Bauer disk diffusion method. Broths from positive blood cultures were subcultured on blood agar, MacConkey’s agar, and XLD agar and then incubated aerobically at 37°C overnight for isolated colonies. These colonies were added to sterile saline solution to make a suspension equivalent to a 0.5 McFarland standard, which was used for antibiotic susceptibility testing. Colonies were also inoculated for biochemical tests. Identification of the organism and susceptibility reporting were completed on the next day, which was approximately 48 hours after the blood culture bottles were flagged as positive. For both methods, antimicrobial susceptibility was reported according to the Clinical and Laboratory Standards Institute’s guidelines and agreements, or errors were compared between test and reference methods [15].

Data analysis

Descriptive analysis of all relevant variables was done by using frequency and percentage. Collected data were checked, and analysis was performed using IBM SPSS Statistics for Windows, Version 20 (Released 2011; IBM Corp., Armonk, New York, United States). Categorical agreement (CA) or "no error": DST and standard AST results agree using the respective criteria. Disagreements were classified as minor errors (mEs), major errors (MEs), and very major errors (VMEs). mEs: Standard method is susceptible (S) or resistant (R) and DST is intermediate (I); alternatively, standard method is intermediate (I) and DST is susceptible (S) or resistant (R). MEs: Standard method yields a susceptible (S) result, whereas DST yields resistance (R). VMEs: Standard method is resistance, and DST is susceptible. CA ≥90% was considered satisfactory [15]. In categorical disagreements, ≤10% mE, <3% ME, and <3% VME were considered acceptable [7,15].

Ethical implications

The study was approved by the ethical committee of the Medical College for Women and Hospital.

## Results

**Figure 1:**
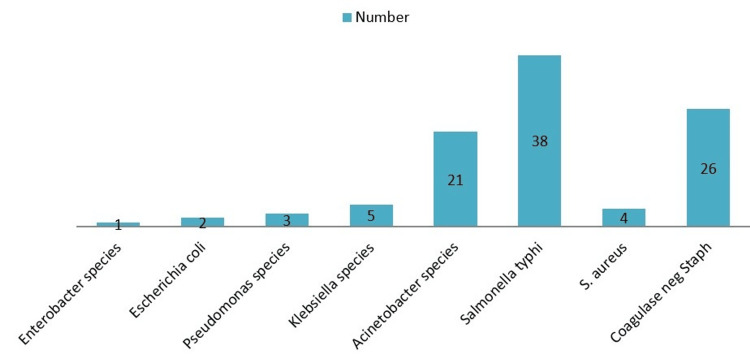
Distribution of bacteria isolated from positive blood cultures (N = 100)

A total of 100 bacteria isolated from the blood culture bottles were compared for DST and standard AST. *Salmonella typhi *(38, 38%) was the most common Gram-negative bacteria, followed by *Acinetobacter* species (21, 21%), *Klebsiella *species (5, 5%), *Pseudomonas* species (3, 3%), *Escherichia coli* (2, 2%) and *Enterobacter *species (1, 1%) (Figure 1). Among Gram-positive bacteria, coagulase-negative *Staphylococci *were the most common (26, 26%), followed by *Staphylococcus aureus (*4, 4%) (Figure 1).

The sensitivity patterns from DST compared to those from standard AST for *Staphylococcus *species are shown in Table 1. Categorical agreement was observed at 96.33% between DST and standard AST, whereas three (1%) mEs, one (0.33%) ME, and seven (2.33%) VMEs were detected. The categorical agreement for ciprofloxacin, levofloxacin, tetracycline, and chloramphenicol was 100%. Most of the VME was detected in azithromycin (13.34%). There was one ME detected for sulfamethoxazole/trimethoprim (3.33%). mEs were found in azithromycin (3.33%), sulfamethoxazole/trimethoprim (3.33%), and gentamicin (3.33%).

**Table 1:** Agreement and error rates between DST and standard AST for Staphylococcus species (n = 30) AST: antimicrobial susceptibility testing

Antibiotic disk (µg)	Categorical agreement n (%)	Categorical disagreement n (%)			Total
		Minor error	Major error	Very major error	
Penicillin (10 µg)	29 (96.67)			1 (3.33)	30
Cefoxitin (30 µg)	29 (96.67)			1 (3.33)	30
Azithromycin (15 µg)	25 (83.33)	1 (3.33)		4 (13.34)	30
Gentamicin (10 µg)	29 (96.67)	1 (3.33)			30
Ciprofloxacin (5 µg)	30 (100)				30
Levofloxacin (5 µg)	30 (100)				30
Sulfamethoxazole/trimethoprim (23.75/1.25 μg)	28 (93.34)	1 (3.33)	1 (3.33)		30
Tetracycline(30 µg)	30 (100)				30
Chloramphenicol(30 µg)	30 (100)				30
Linezolid (30 µg)	29 (96.67)			1 (3.33)	30
Overall agreement	289 (96.33)	3 (1)	1(0.33)	7 (2.33)	300

Table 2 showed 95.19% categorical agreement between DST and standard AST for *Escherichia coli*, *Klebsiella *species, and *Enterobacter *species. mEs, MEs, and VMEs were found at 0.96%, 2.89%, and 0.96%, respectively. One mE for ceftriaxone, three MEs each for ceftazidime, ceftriaxone, and tetracycline, and one VME for piperacillin-tazobactam were found.

**Table 2:** Agreement and error rates between DST and standard AST for Escherichia coli, Klebsiella species, and Enterobacter species (n = 8) DST: direct susceptibility testing; AST: antimicrobial susceptibility testing

Antibiotic disk (µg)	Categorical agreement n (%)	Categorical disagreement n (%)			Total
		Minor error	Major error	Very major error	
Amoxicillin-clavulanate (20/10 µg)	8 (100)				8
Piperacillin-tazobactam (100/10 µg)	7 (87.5)			1 (12.5)	8
Cefuroxime (30 µg)	8 (100)				8
Ceftazidime (30 µg)	7 (87.5)		1 (12.5)		8
Ceftriaxone (30 µg)	6 (75)	1 (12.5)	1 (12.5)		8
Cefixime (5 µg)	8 (100)				8
Meropenem (10 µg)	8 (100)				8
Gentamicin (10 µg)	8 (100)				8
Amikacin (30 µg)	8 (100)				8
Ciprofloxacin (5 µg)	8 (100)				8
Levofloxacin (5 µg)	8 (100)				8
Tetracycline (30 µg)	7 (87.5)		1 (12.5)		8
Sulfamethoxazole/trimethoprim (23.75/1.25 μg)	8 (100)				8
Overall agreement	99 (95.19)	1 (0.96)	3 (2.89)	1 (0.96)	104

Piperacillin-tazobactam was found. Out of 304 antimicrobial agent combinations for *Escherichia typhi*, 290 (95.4%) showed categorical agreement (Table 3). In addition, four (1.32%) mEs were detected for ciprofloxacin, and five (1.64%) MEs were found in azithromycin and ciprofloxacin. Among five (1.64%) VMEs, most were in tetracycline (5.26%), followed by ampicillin, cefixime, and meropenem.

**Table 3:** Agreement and error rates between DST and standard AST for S. typhi (n = 38) DST: direct susceptibility testing; AST: antimicrobial susceptibility testing

Antibiotic disk (µg)	Categorical agreement n (%)	Categorical disagreement n (%)			Total
		Minor error	Major error	Very major error	
Ampicillin (10 µg)	37 (97.37)			1 (2.63)	38
Ceftriaxone (30 µg)	38 (100)				38
Cefixime (5 µg)	37 (97.37)			1 (2.63)	38
Ciprofloxacin (5 µg)	32 (84.21)	4 (10.52)	2 (5.26)		38
Sulfamethoxazole/trimethoprim (23.75/1.25 μg)	38 (100)				38
Azithromycin (15 µg)	35 (92.1)		3 (7.89)		38
Tetracycline (30 µg)	36 (94.74)			2 (5.26)	38
Meropenem (10 µg)	37 (97.37)			1 (2.63)	38
Overall agreement	290 (95.4)	4 (1.32)	5 (1.64)	5 (1.64)	304

Categorical agreement for DST and standard AST was found to be 94.70% for *Acinetobacter *species. mEs, MEs, and VMEs were found at 3.17%, 1.6%, and 0.53%, respectively. mEs for sulfamethoxazole/trimethoprim (19.04%), ceftriaxone (4.76%), and piperacillin-tazobactam (4.76%) and MEs for ciprofloxacin (9.52%) and levofloxacin (4.76 %) were found. One VME was detected in meropenem (4.76%) (Table 4).

**Table 4:** Agreement and error rates between DST and standard AST for Acinetobacter (n = 21) DST: direct susceptibility testing; AST: antimicrobial susceptibility testing

Antibiotic disk (µg)	Categorical agreement n (%)	Categorical disagreement n (%)			Total
		Minor error	Major error	Very major error	
Piperacillin-tazobactam (100/10 µg)	20 (95.24)	1 (4.76)			21
Ceftazidime (30 µg)	21 (100)				21
Ceftriaxone (30 µg)	20 (95.24)	1 (4.76)			21
Meropenem (10 µg)	20 (95.24)			1 (4.76)	21
Gentamicin (10 µg)	21 (100)				21
Amikacin (30 µg)	21 (100)				21
Ciprofloxacin (5 µg)	19 (90.48)		2 (9.52)		21
Levofloxacin (5 µg)	20 (95.24)		1 (4.76)		21
Sulfamethoxazole/trimethoprim (23.75/1.25 μg)	17 (80.95)	4 (19.04)			21
Overall agreement	179 (94.70)	6 (3.17)	3 (1.6)	1 (0.53)	189

In the case of *Pseudomonas *species, neither a very major nor a major error was seen. Categorical agreement was found at 93.33% and one mE (6.67%) was detected in ciprofloxacin (Table 5).

**Table 5:** Agreement and error rates between DST and standard AST for Pseudomonas (n = 3) DST: direct susceptibility testing; AST: antimicrobial susceptibility testing

Antibiotic disk (µg)	Categorical agreement n (%)	Categorical disagreement n (%)			Total
		Minor error	Major error	Very major error	
Piperacillin-tazobactam (100/10 µg)	3 (100)				3
Ceftazidime (30 µg)	3 (100)				3
Ciprofloxacin (5 µg)	2 (66.67)	1 (33.33)			3
Levofloxacin (5 µg)	3 (100)				3
Meropenem (10 µg)	3 (100)				3
Overall agreement	14 (93.33)	1 (6.67)	0(0)	0(0)	15

## Discussion

Life-threatening sepsis caused by pathogenic bacteria demands prompt antimicrobial treatment. Early identification of causative agents and their antibiotic sensitivity is crucial for improving patient outcomes. Direct sensitivity testing provides results within 18-24 hours after a positive blood culture signal, significantly faster than standard AST results, which take 36-48 hours. This study aimed to compare the direct AST with the standard disk diffusion testing from the positive blood culture bottle flagged by the automated blood culture system. The DST results were compared with the standard AST results.

In this study, a total of 100 bacterial isolates were included from positive blood cultures. Among Gram-positive bacteria, coagulase-negative *Staphylococci *were the most common (26%), followed by *Staphylococcus aureus* (4%). Similar results were reported by studies conducted in India and the United States [4,16]. However, *Staphylococcus aureus *(44%) was found as the most common microorganism in a study done in Spain [17].

In the present study, *Staphylococcus *species showed 96.33% categorical agreement with 1% mEs, 0.33% MEs, and 2.33% VMEs between DST and AST. Similar categorical agreements have been reported by other studies in India and the United States, where CA was 99.2%, 98%, and 91% [3,4,18]. Several studies have reported different error rates for different antibiotic panels. In our study, an ME was detected for penicillin, which is consistent with another study in India [8]. However, cefoxitin was categorized in VME in our findings, while other studies mentioned it in an ME [3,8].

*Escherichia coli*, *Klebsiella *species, and *Enterobacter *species showed 95.19% categorical agreement in the present study. Similar findings were also observed by the authors, where CA was 95.6% and 96.1%, respectively [3,8]. Piperacillin-tazobactam showed a VME in the current study. Several studies also reported VMEs, MEs, and mEs for this drug [4,8,13,19]. Similar to another report, MEs and mEs were detected for ceftriaxone in this study [8].

*Salmonella typhi *showed 95.4% categorical agreement with 1.32% mEs, 1.64% MEs, and 1.64% VMEs. Similar to this study, 100% CA between both methods for cotrimoxazole and ceftriaxone, with mEs in ciprofloxacin, was found by another study in Pakistan [20]. The study showed categorical agreement of 94.70% for *Acinetobacter *species and 93.33% for *Pseudomonas *species, with 3.17% mEs, 1.6% MEs, and 0.53% VMEs for *Acinetobacter *species, while 6.67% mEs in *Pseudomonas* species. Similar categorical agreements were observed by research done in Malaysia and India [7,8].

The current study showed that overall categorical agreement, mEs, MEs, and VMEs in each group of organisms were within acceptable limits. Individual antibiotic performance was mostly within or slightly below acceptable limits. However, marginally high VME rates were observed for azithromycin against *Staphylococcus *spp. (13.34%), meropenem against *Acinetobacter *spp. (4.76%), tetracycline against *Salmonella typhi* (5.26%), and piperacillin-tazobactam against *Escherichia coli*, *Klebsiella*, and *Enterobacter *species.

Discrepancies between direct and standard AST methods are caused by multiple factors. These include inoculum effect (too heavy or too low), variations in filter paper disc potency (even within the same vial), quality of the growth media, and incubation conditions (temperature, humidity, power supply). Each of these can lead to inaccurate microorganism growth and erroneous test results [13]. Despite major advancements in rapid susceptibility testing, many automated methods remain inaccessible in low to middle-income countries where AMR emerges and spreads.

Direct sensitivity testing offers a significant advancement, providing results in 18-24 hours, which is considerably faster than standard AST. DST can be set up in a routine diagnostic laboratory. This accelerated diagnostic approach enables clinicians to implement targeted treatments more quickly and reduce complications associated with inappropriate antibiotic use.

This study is not beyond limitations. This is a single-centred study, and the sample size is not enough to draw a strong, conclusive decision. In the future, a multi-centred study with more samples can be done to overcome these limitations. A future large-scale study can be done with the help of the minimum inhibitory concentration (MIC) method to obtain more accurate and precise results.

## Conclusions

The performance of direct sensitivity testing from positive blood culture demonstrated acceptable error levels across various organism groups in antimicrobial susceptibility testing, although it slightly exceeded the acceptable thresholds. DST serves as an available alternative in resource-limited settings, providing results more quickly than traditional AST and facilitating the prompt initiation of appropriate antibiotics.
